# How gut microbiota contribute to neuropsychiatric disorders: evidence from neuroimaging studies

**DOI:** 10.3389/fmicb.2026.1760096

**Published:** 2026-03-04

**Authors:** Chunlan Jia, Wenjie Zhu, Yanling Yuan, Qinglian Xie

**Affiliations:** 1 Center for Neurological Function Testing and Regulation, West China Xiamen Hospital, West China Hospital, Sichuan University, Xiamen, Fujian, China; 2 West China School of Medicine, Sichuan University, Chengdu, China; 3 Department of Pharmacy, West China Hospital, Sichuan University, Chengdu, China; 4 Department of Outpatient, West China Xiamen Hospital, West China Hospital, Sichuan University, Chengdu, China

**Keywords:** Alzheimer’s disease, autism spectrum disorder, central nervous system diseases, depression, gut microbiota, microbiota-gut-brain axis, multiple sclerosis, neuroimaging

## Abstract

The interaction between the gut microbiota and central nervous system (CNS) diseases has emerged as a major focus in neuroscience and microbiome research. Accumulating evidence shows that gut microbiota influence the pathogenesis of neurodevelopmental, neurodegenerative, autoimmune, and psychiatric conditions via the microbiota-gut-brain axis. However, the underlying mechanisms are complex and not yet fully elucidated. Advances in multimodal magnetic resonance imaging, positron emission tomography, and diffusion tensor imaging, now enable *in vivo* visualization of associations between gut microbial alterations and abnormalities in brain structure and function, providing new perspectives for understanding the role of gut microbiota in CNS pathology. This review systematically reviews neuroimaging-based research linking gut microbiota to neurological diseases (e.g., Alzheimer’s disease, multiple sclerosis, traumatic brain injury), and psychiatric disorders (e.g., schizophrenia, and autism spectrum disorder). It highlights the mediating roles of microbial metabolites, immune-inflammatory responses, and neuroimmune pathways, and discusses future directions integrating multi-omics data with neuroimaging technologies, as well as their potential clinical applications. What distinguishes this review from its predecessors in the same field is its explicit neuroimaging-driven framework rather than general mechanistic discussion.

## 1 Introduction

The gut microbiota, a complex and dynamic symbiotic ecosystem, plays a crucial role in maintaining host physiological homeostasis, influencing metabolic, immune, as well as the health and function of the central nervous system (CNS) (Shaik et al., 2020; Shekarabi et al., 2024). Recent evidence that gut microbiota communicate bidirectionally with the CNS through the microbiota–gut–brain axis (MGBA) has reshaped our understanding of brain health and disease. This integrating network, involving neural, endocrine, and immune pathways, has become a central focus of interdisciplinary research (Cheng et al., 2024; Longo et al., 2023). As research across neurodevelopmental, neurodegenerative, inflammatory, and psychiatric disorders expands, it is increasingly evident that gut-derived signals significantly influence CNS function. However, the precise mechanisms by which gut microbiota influence CNS function, and how these effects are detected in the living human brain, remain incompletely understood.

Among these mechanisms, gut microbial metabolites, including short-chain fatty acids (SCFAs), tryptophan metabolites, and lipopolysaccharide (LPS), are core mediators of gut–brain communication and play a central role in modulating intestinal barrier integrity, blood–brain barrier (BBB) permeability, and neuroinflammation (Cheng et al., 2024; Liu Y. et al., 2025; Sadler et al., 2020; Shekarabi et al., 2024; Stolzer et al., 2023; Yousefi et al., 2022). These molecules influence microglial activation, synaptic plasticity, and neurotransmitter regulation, thereby directly shaping neural structure and function. For instance, SCFAs can cross the BBB to influence neuronal and glial cell function, exerting significant regulatory effects in disease models such as depression, multiple sclerosis, and post-stroke neural repair (Cheng et al., 2024; Sadler et al., 2020; Yousefi et al., 2022). Moreover, multiple studies have indicated that individual factors, such as sex, age, genetic polymorphism (e.g., APOE genotype), and dietary habits, contribute to variability in microbiota–CNS interactions, further highlighting the complexity of these regulatory networks (Randeni and Xu, 2025; Zhang et al., 2023; Zhang Z. et al., 2021). These findings highlight the need for *in vivo* approaches capable of capturing how microbial signals translate into brain-level outcomes.

Given these multifaceted mechanisms, the rapid development of neuroimaging techniques, such as magnetic resonance imaging (MRI), functional MRI (fMRI), diffusion tensor imaging (DTI), and positron emission tomography (PET) allow non-invasive characterization of brain microstructure, functional network dynamics, metabolic patterns, and neuroinflammatory activity (Montoro et al., 2022; Yi et al., 2022; Zhang et al., 2022). Structural MRI and DTI can detect gray matter atrophy and impairment of white matter integrity; fMRI can reflect abnormal functional connectivity in core brain networks such as the default mode network and prefrontal network; PET can quantify cerebral metabolism, β-amyloid deposition, and neuroinflammation levels. Together, these techniques directly link peripheral microbial changes to central abnormalities in structure, function, and metabolism, providing visual evidence for deciphering the role of the MGBA in neuropsychiatric disorders. Some neuroimaging studies have linked microbial diversity and composition with multimodal neuroimaging alterations in key brain networks, involving the default mode network, visual, and prefrontal networks, as well as cognitive and emotional outcomes (Zhang et al., 2022; Zhu et al., 2022). Research on healthy populations further demonstrates associations between gut microbial composition and brain phenotypes, including neuroanatomy, functional network connectivity (e.g., Default Mode Network), sleep, working memory, and attention, and related cognitive and behavioral outcomes (Bonham et al., 2023; Cai et al., 2021; Graf et al., 2025; Querdasi et al., 2025). Importantly, gut–brain interactions appear to emerge early in life: neonatal studies show connections between microbial profiles and brain regions supporting emotion, sensation, and interoception (Graf et al., 2025; Ramadan et al., 2025). And the early-life microbial profiles (2-year old) are associated with subsequent internalizing symptoms like anxious/depressed, withdrawn/depressed, and somatic complaints, suggesting that gut–brain interactions are established from infancy and influence developmental trajectories (Querdasi et al., 2025). These findings illustrate the potential of neuroimaging to translate peripheral microbial signals into measurable central neural outcomes.

Clinical research similarly supports the role of MGBA alterations in disease. Gut microbial dysbiosis correlates with structural atrophy and functional network disruptions in disorders such as Alzheimer’s disease (AD) and multiple sclerosis (MS) (Liu Y. et al., 2025; Padhi et al., 2022; Wu et al., 2025a). Studies integrating neuroimaging with microbiome profiling have begun to outline disease-relevant gut–brain pathways, offering promising avenues for precision and personalized medicine (Ge et al., 2025; Loh et al., 2024). Nevertheless, most work to date has examined single disorders or relied on isolated imaging modalities, limiting broader insights into shared versus disease-specific MGBA signatures.

These gaps underscore the need for a systematic synthesis integrating microbial mechanisms with multimodal neuroimaging findings across diverse neuropsychiatric conditions. Accordingly, this review aims to: (1) summarize key microbial signals and biological mechanisms relevant to CNS function; (2) integrate multimodal neuroimaging findings associated with microbiota alterations; (3) compare disease-specific and cross-disorder patterns; and (4) propose a conceptual framework linking gut microbial perturbations to CNS imaging phenotypes. By combining mechanistic insights with neuroimaging and microbiome evidence, this review provides a structural and integrative perspective that is expected to accelerate the elucidation of MGBA pathways and support advances in precision diagnosis and treatment strategies (Ge et al., 2025; Montoro et al., 2022; Silva et al., 2020).

## 2 The microbiota-gut-brain axis

The MGBA represents a bidirectional communication network between the gut microbiota and the CNS. The MGBA integrates multiple mechanistic pathways, involving neural signaling, immune regulation, microbial metabolite, and neuroendocrine pathways, to maintain neuropsychiatric health and multi-system homeostasis.

Microbial metabolites constitute one of the most direct biochemical routes linking the gut to the brain. SCFAs regulate neuronal and glial cell activity by influencing BBB integrity, modulating neuroinflammatory signaling, and influencing neurotransmitter synthesis. For example, butyrate is considered as a histone deacetylase inhibitor that reshapes gene expression in neurons, thereby affecting cognition, mood, and stress response (Cheng H. et al., 2025; Silva et al., 2020). Similarly, tryptophan metabolites, such as kynurenic acid and dopamine, interact with neuroprotective signaling pathways (e.g., NFE2L2/NRF2), regulating oxidative stress responses and neural repair processes in the CNS (Liu H. X. et al., 2025). These metabolites also regulate neurotransmitter systems, thereby regulating sleep, mood, and cognitive function (Cheng H. et al., 2025; Han et al., 2022). These findings highlight microbial metabolites as central mediators that bridge peripheral metabolic states with CNS function.

The gut microbiota plays a crucial role in regulating host immune homeostasis. By maintaining intestinal barrier integrity, it restricts harmful substances and pathogens from entering the bloodstream, thus preventing peripheral inflammation. When dysbiosis disrupts this barrier, circulating inflammatory mediators like lipopolysaccharide (LPS) gain access to the bloodstream, activating immune responses and inducing neuroinflammatory processes within the CNS. These mechanisms contribute to the pathological processes of neuropsychiatric diseases, such as Alzheimer’s disease, multiple sclerosis, and schizophrenia (Menees et al., 2022; Pellegrini et al., 2020; Talat et al., 2025). Furthermore, cytokines and chemokines produced by gut-associated immune cells can modulate the brain’s immune environment via hematogenous or neural routes. By regulating microglial activation, these peripheral immune signals modulate key processes—including neuroinflammation, neurodegeneration, and synaptic remodeling—that ultimately impact behavioral outcomes and disease progression (Balakrishnan et al., 2024; Dworsky-Fried et al., 2020).

The integrity of both the intestinal barrier and the BBB is essential for maintaining CNS stability. Microbial metabolites, immune mediators, and dysbiosis-induced inflammation can modulate tight-junction protein expression, thereby altering permeability in both systems (Braniste et al., 2014; Fung et al., 2017). Increased intestinal permeability facilitates the systemic spread of microbial products, while BBB disruption enhances the entry of circulating cytokines and immune cells into the CNS, amplifying neuroinflammation and neuroimmune dysregulation (Banks, 2016). Such bidirectional barrier disruption creates a feed-forward loop implicated in neuropsychiatric and neurodegenerative conditions (Cryan et al., 2019; Kelly et al., 2015). Although less frequently emphasized, recent findings indicate that microbiota-dependent regulation of peripheral immunity may also promote the entry of immune cells into the CNS, providing a complementary mechanism within the broader MGBA framework (White et al., 2025).

At the neural pathway level, the vagus nerve and the enteric nervous system (ENS) constitute the primary neural bridge of the MGBA. The vagus nerve senses changes in the gut environment and transmits signals to the brainstem, regulating neuroendocrine and immune responses. Additionally, the ENS, the “second brain,” not only regulates gastrointestinal motility and secretion, but also interacts with the CNS to influence emotional regulation, stress responses, and behavior (Bistoletti et al., 2020; Silva et al., 2020). Additionally, gut microbiota and their metabolites modulate neurotransmission by producing or regulating precursors of key neurotransmitters, such as serotonin (5-HT) and gamma-aminobutyric acid (GABA). These molecules shape ENS activity and, through neuroimmune and neural pathways, exert broader effects on brain function and behavior (Chen et al., 2021; Miri et al., 2023). Furthermore, microbial metabolites also influence neuroplasticity through modulation of synaptic proteins, neurotrophic factors, and intracellular signaling cascades.

Beyond the main pathways mentioned above, the MGBA further interacts with neuroendocrine system, epigenetic regulation, and lipid metabolism. For instance, the gut microbiota regulates endocrine hormones like incretins (GLP-1), peptide YY (PYY), and cholecystokinin (CCK), affecting appetite, energy metabolism, and mood states (Kasarello et al., 2023; Wijdeveld et al., 2020). Epigenetically, metabolites like butyrate can regulate DNA methylation, histone modification, and non-coding RNA expression, thereby regulating neuronal development and vulnerability to mood disorders (Begum et al., 2022; Liu et al., 2023). In lipid metabolism pathways, microbially driven bile acid metabolism and unsaturated fatty acids, such as palmitoleic acid, regulate neuroinflammation and neuroprotection, showing increasingly relevance in neurodegenerative diseases like Alzheimer’s disease (Chen et al., 2024).

In summary, the MGBA works as a multi-level, multi-pathway communication network through which gut microbiota modulate CNS function and contribute to the onset and progression of neuropsychiatric disorders. By microbial metabolites, immune regulation, barrier integrity, neural transmission, and neuroendocrine pathways, the gut microbiota exerts profound effects on brain structure, function, and behavior. As research deepens, these mechanisms provides a theoretical basis and practical possibilities for microbiota-targeted intervention strategies (Ghezzi et al., 2022; Sittipo et al., 2022; Talat et al., 2025).

## 3 Application of neuroimaging techniques in neuropsychiatric disorders

Neuroimaging has been widely used to characterize brain alterations in neuropsychiatric disorders. Multimodal MRI, combining structural MRI, fMRI, and DTI, allows for a comprehensive assessment of regional brain morphology, white-matter integrity, and functional connectivity (FC). These techniques have been widely used in schizophrenia, depression, and Alzheimer’s disease, to identify pathological brain regions and network abnormalities, and assist in early diagnosis and subtyping (Elias-Mas et al., 2021; Vacca et al., 2022; Wen et al., 2024).

Functional imaging techniques further enhance this characterization. 18F-FDG positron emission tomography (FDG-PET) can quantify cerebral glucose metabolism, reflecting neuronal functional status. Regional hypometabolism detected by FDG-PET relates to cognitive deficits in conditions like Alzheimer’s and Parkinson’s disease (Martín-Bastida et al., 2021; Vacca et al., 2022). Furthermore, quantitative susceptibility mapping (QSM) and T2* imaging provide non-invasive tool for the quantifying brain iron load, which is implicated in various neuropsychiatric and neurodegenerative pathologies. These techniques facilitate the analysis of how gut microbiota influence brain iron load, providing imaging evidence for MGBA metabolism (Mandal et al., 2023; Wang S. et al., 2023).

Integrating multimodal neuroimaging with omics (microbiome and metabolomics) data provides new approaches for multi-dimensional exploration of gut-brain interactions. Multi-omics data fusion can reveal associations between microbial metabolites and changes in brain structure, function, and metabolism, promoting understanding of the complex pathological networks. Utilizing artificial intelligence (AI) and machine learning for deep integration of imaging and omics data has become a key direction for precision medicine, advancing early diagnosis, disease subtyping, and precision interventions in neuropsychiatric disorders (Huang and Shu, 2025; Wang Y. et al., 2024; Wen et al., 2024).

## 4 Potential mechanisms of gut microbiota influencing brain imaging changes

### 4.1 Microbial metabolites

Gut microbiota produce various bioactive molecules through metabolism, particularly SCFAs and tryptophan metabolites, which influence brain structure and function, primarily by regulating BBB permeability and neuroinflammatory responses. SCFAs, including acetate, propionate, and butyrate, are crucial for maintaining intestinal barrier integrity, regulating immune responses, and supporting CNS homeostasis (Ravi et al., 2025). They also enhance BBB structural integrity, promote the expression of tight junction proteins, restricting peripheral inflammatory signals from entering the CNS (Knox et al., 2022). Additionally, SCFAs exert neuroprotective effects through inhibiting histone deacetylase activity and regulating gene expression in neurons and glial cells (Ravi et al., 2025). In neurodegenerative diseases like Parkinson’s disease (PD), abnormal SCFA levels correlate with disease progression, suggesting their potential therapeutic value (Ran et al., 2025; Ravi et al., 2025). Tryptophan metabolites also participate in regulating brain function. Specifically, indole compounds mediated by gut microbiota, such as indole-3-acetic acid (IAA), can cross the BBB, modulate neurotransmitter systems and neuroendocrine signaling pathways, thereby affecting mood and cognitive performance (Chernevskaya et al., 2021; Yu et al., 2024). Furthermore, abnormalities in tryptophan metabolic pathways involve in the pathology of various neuropsychiatric disorders, including depression and autism spectrum disorder (Więdłocha et al., 2021; Wiley et al., 2021). Many factors like certain food components (e.g., artificial sweeteners) can influence this physiological process, which characterized by a reduction in beneficial bacteria and an increase in pathogenic bacteria, thereby affecting the production of SCFAs and ultimately impacting BBB function, neuroinflammatory responses, and brain function (Hetta et al., 2025b).

**Figure 1:**
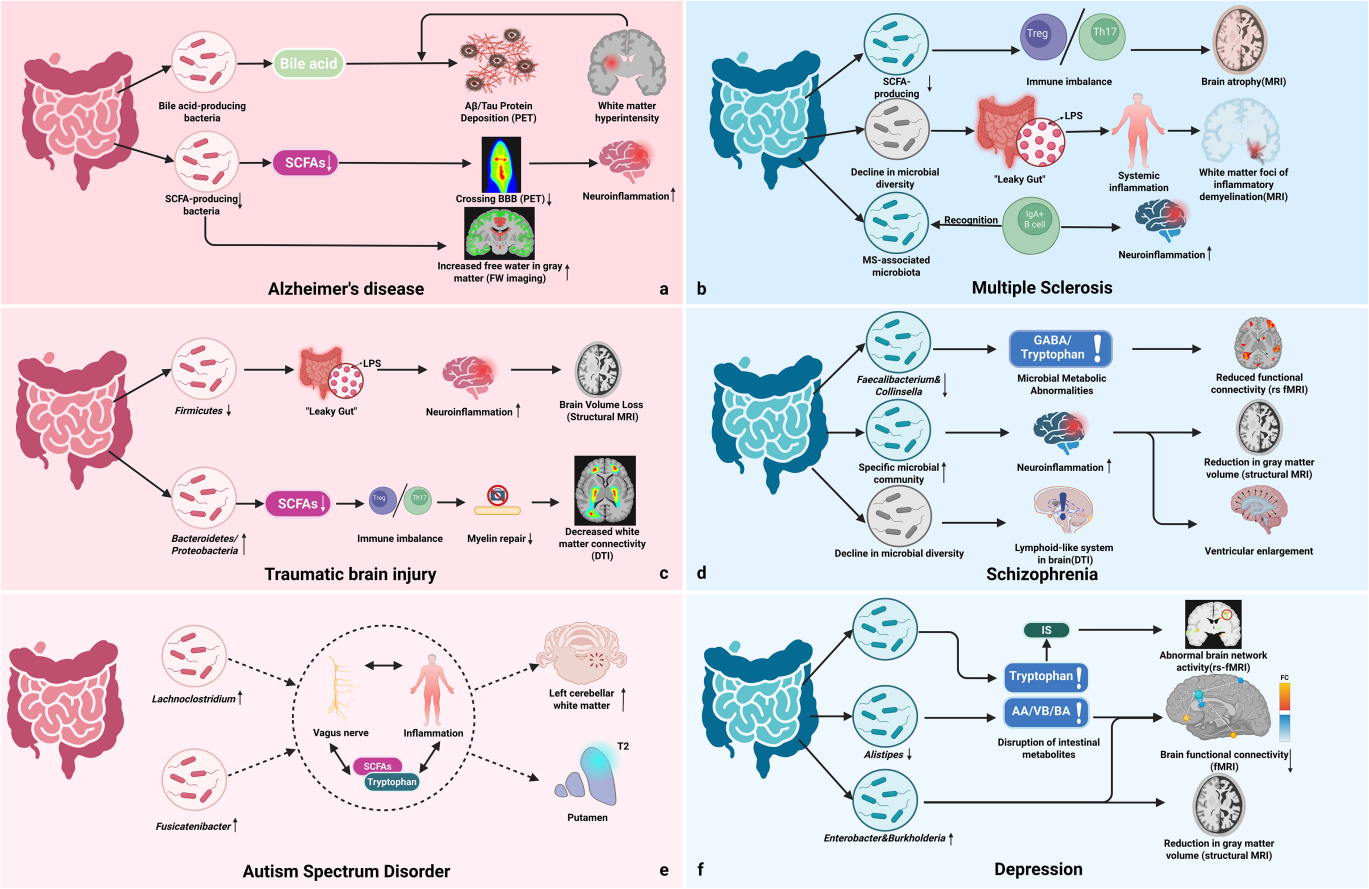
Neuroimaging evidence linking gut microbiota to neuropsychiatric disorders (Davis et al., 2022; Davis Iv et al., 2025; Tsai et al., 2024; Yamashiro et al., 2024; Zhao Y. et al., 2025). **(a)** Alzheimer’s disease (AD): dysbiosis (SCFA↓, Bile Acid metabolism) correlates with core pathologies: Aβ/Tau deposition (PET), impaired BBB integrity (PET), increased neuroinflammation, and structural changes (e.g., increased gray matter free water, FW imaging). **(b)** Multiple sclerosis (MS): reduced SCFA producers contribute to Th17/Treg imbalance and observable brain atrophy (MRI). Reduced diversity of the microbiota facilitates LPS-induced systemic inflammation, correlating with inflammatory demyelinating lesions in white matter (T2/FLAIR MRI). **(c)** Traumatic brain injury (TBI): altered bacterial ratios (e.g., *Firmicutes*↓) induce intestinal permeability, leading to gray matter volume reduction or impaired white matter connectivity (DTI) via immune dysregulation. **(d)** Schizophrenia: decreased specific genera (e.g., *Faecalibacterium* and *Collinsella*) are associated with reduced functional connectivity (rs-fMRI). Inflammation or impaired Glymphatic system function (DTI data) may drive ventricular enlargement and gray matter volume loss. **(e)** Autism spectrum disorders (ASD): changes in specific genera (e.g., *Lachnoclostridium*, *Fusicatenibacter*) correlate with white matter volume (DTI) and T2 signal alterations, suggesting microbial influence on myelin formation. **(f)** Depression: microbial alterations (e.g., *Alistipes*↓) disrupt AA, VB, and BA metabolism, linking to decreased functional connectivity (fMRI) and structural changes (MRI). Tryptophan metabolites (e.g., IS) may activate aversive processing networks (rs-fMRI) linked to anxiety. SCFAs, short-chain fatty acids; PET, positron emission tomography; FW, free water; LPS, lipopolysaccharide; MRI, magnetic resonance imaging; DTI, diffusion tensor imaging; rs-fMRI, resting-state functional magnetic resonance imaging; AA, amino acid; VB, vitamin B; BA, bile acid; IS, indole-3-pyrothioic acid; fMRI, functional magnetic resonance imaging; FC, functional connectivity.

Multi-omics and neuroimaging studies further confirm the close associations between microbial metabolites and FC in cognitive and emotional networks. Structural changes in gut microbiota and their metabolites correlate with the pathogenesis of various conditions, including cognitive decline and dementia, influencing gray matter volume and white matter integrity (Alloo et al., 2022; Hsu et al., 2025; Liang et al., 2022). Specifically, the abundance of SCFAs and tryptophan metabolites positively correlates with hippocampal volume and prefrontal cortex FC (Dong et al., 2020; Liang et al., 2022). Furthermore, 7T MRI results revealed that SCFA supplementation significantly alleviated hippocampal atrophy, and correlated with increases in beneficial gut microbiota such as *Alloprevotella* (Lee P. J. et al., 2025). Clinical intervention studies further supports this regulatory role, and they found that modulation of gut microbiota in obese patients improved connectivity in cognitive-related brain networks (Dong et al., 2020; Xiang et al., 2024). Figure 1 summarizes key neuroimaging findings linking microbial metabolites (e.g., SCFAs and tryptophan derivatives) to alterations in BBB integrity, neuroinflammation, and brain structure and function across CNS disorders.

### 4.2 Immune-inflammatory pathway

Gut microbiota influence microglial activation and neuroinflammation in the CNS by regulating peripheral immune responses, leading to brain structural and functional changes. Multiple studies indicate that peripheral inflammatory signals can cross the BBB and alter the CNS immune milieu, driving microglial activation and neuroinflammatory cascades (Jung et al., 2022). Microbial metabolites and immunomodulatory effects similarly affect neuroinflammation and regulate neuronal survival and synaptic function.

Brain imaging provides quantitative markers of these immune-related effects. MRI DTI shows that peripheral inflammatory status correlates with microstructural white matter abnormalities, such as increased diffusivity and altered axial diffusivity, reflecting axonal damage and demyelination (Lan et al., 2024). In PET imaging, specific radioligands for neuroinflammation markers, such as translocator protein (TSPO, e.g., [18F]GE-180 and [11C]CPPC) and GPR84 ligands, can monitor microglial and myeloid cell activation (Kalita et al., 2023; Lee S. J. et al., 2025; Lucot et al., 2022).

Integration of immune markers and neuroimaging further reveals disease progression mechanisms. Peripheral inflammatory markers like C-reactive protein (CRP) and systemic immune-inflammation index (SII) closely correlate with reduced gray and white matter volume, with brain atrophy partially mediating the effect of systemic inflammation on cognitive decline (Jung et al., 2022; Zhuo et al., 2023). Cytokines, such as egulated upon activation normal T cell expressed and secreted (RANTES), Hepatocyte growth factor (HGF) and IL-13, serve as mediators of the gut-brain axis, influencing the structural connectivity between brain regions (Guo et al., 2025). Simultaneously, abnormal brain FC show negative correlation with peripheral inflammatory markers, suggesting the key role of immune dysregulation in CNS network impairment (Aruldass et al., 2021). In psychiatric disorders such as schizophrenia and depression, immune-inflammatory subtypes identified through functional imaging contribute to more refined disease stratification (Bishop et al., 2022; Day et al., 2021).

**Figure 2:**
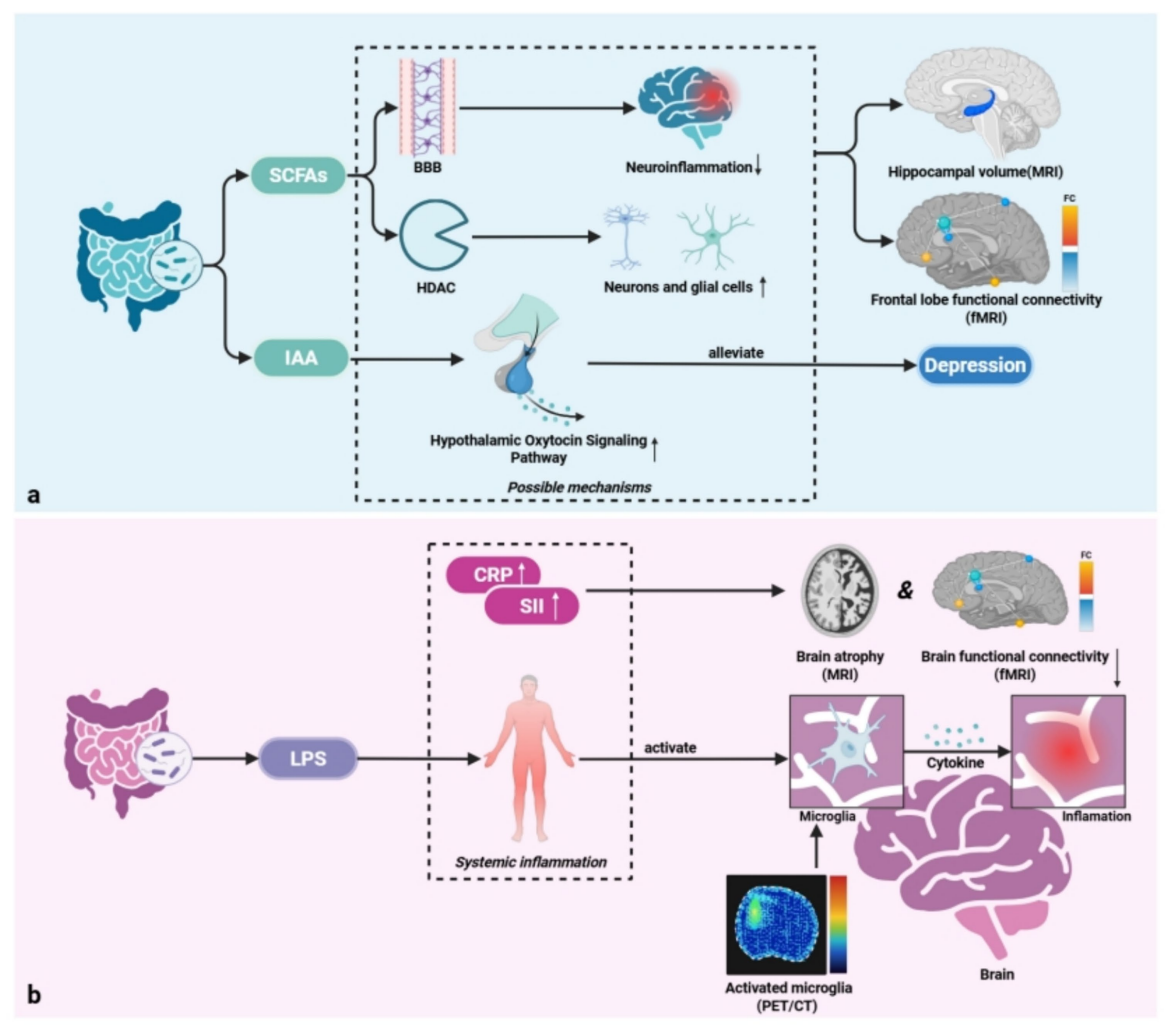
Potential mechanisms by which gut microbiota influence brain imaging changes (Lucot et al., 2022). **(a)** Microbial metabolite pathways: key metabolites (SCFAs, IAA) exert neuroprotective effects by maintaining BBB integrity, reducing neuroinflammation, and modulating neuronal gene expression. They show positive correlations with structural (e.g., hippocampal volume, MRI) and functional (e.g., prefrontal cortex connectivity, fMRI) imaging markers. **(b)** Immune-inflammatory pathways: LPS induces systemic inflammation, leading to microglia activation (observed via PET/CT) and subsequent neuroinflammation. Peripheral inflammatory markers (CRP, SII) correlate with adverse neuroimaging outcomes, including brain atrophy (MRI) and reduced functional connectivity (fMRI). SCFAs, short-chain fatty acids; IAA, indole-3-acetic acid; BBB, blood–brain barrier; HDAC, histone deacetylase; MRI, magnetic resonance imaging; fMRI, functional magnetic resonance imaging; FC, functional connectivity; LPS, lipopolysaccharide; CRP, C-reactive protein; SII, systemic immune inflammation index; PET/CT, positron emission tomography-computed tomography. Italicized terms summarize the content within dashed boxes.

Furthermore, meningeal inflammation, an important site for CNS immune surveillance, reveals strong spatial transcriptomics links to inflammatory gene expression patterns in adjacent brain parenchyma, highlighting its influence on local pathological status (Gadani et al., 2024). Advanced imaging technologies, including two-photon microscopy and immune cell-specific nanoprobes, allow dynamic, high-resolution observation of brain immune cells and real-time monitoring of neuroinflammatory processes (Byun et al., 2022; Wang X. et al., 2023). Figure 2 provides a comprehensive insight into how microbial metabolites and immune-inflammatory signaling interact and influence neuroimaging phenotypes.

## 5 Neuroimaging evidence for the association between gut microbiota and neuropsychiatric disorders

This review covers a broad spectrum of neuropsychiatric disorders, encompassing neurological diseases (e.g., Alzheimer’s disease, multiple sclerosis, traumatic brain injury) and psychiatric disorders (e.g., schizophrenia, autism spectrum disorder). Accumulating evidence has confirmed that the onset and progression of these conditions are directly linked to gut microbiota. As summarized in Supplementary Table S1, we systematically compare the gut microbiota-associated neuroimaging signatures across these disorders, providing a foundational reference for the subsequent disease-specific analysis of neuropsychiatric disorders.

### 5.1 Alzheimer’s disease

Alzheimer’s disease (AD), the most common neurodegenerative disorder, is characterized by β-amyloid (Aβ) accumulation, tau hyperphosphorylation, and progressive cognitive decline (Ivanova et al., 2025; Pluta et al., 2020). Recently, increasing evidence suggests that gut microbiota contribute to AD pathology by inflammatory, metabolic, and neuroimmune mechanisms (Borsom et al., 2020; Sheng et al., 2023). Combining neuroimaging evidence with microbiome data provides a new perspective for revealing these interactions.

Previous studies show that gut dysbiosis is closely associated with brain structural atrophy and metabolic abnormalities in AD patients. APP/PS1 transgenic mice exhibit cognitive impairment accompanied by peripheral organ metabolic disturbances, significant changes in fecal metabolites, and marked alterations in gut microbiota composition, suggesting a correlation between gut microbiota, peripheral metabolic status, and brain pathology (Li et al., 2025). Furthermore, multimodal MRI and diffusion kurtosis imaging (DKI) demonstrate that microstructural changes and elevated neuroinflammation markers co-occurred with significantly reduced gut microbiota diversity and lower SCFA content, further linking abnormal gut microbial metabolic function to AD progression (Palanivelu et al., 2025). Human studies also observed consistent association that gray and white matter damage in AD and mild cognitive impairment (MCI) is negatively correlated with the abundance of butyrate-producing genera (e.g., *Anaerostipes*, *Ruminococcus*) (Yamashiro et al., 2024). And lower Bacteroidetes abundance was associated with greater Aβ deposition as confirmed by PET results (Kojima et al., 2025).

Integration of radiomics and microbiomics reveals the regulatory mechanisms of microbial metabolites on brain function. Combining 16S rRNA sequencing, serum and fecal metabolomics, and PET/MRI imaging found that altered microbial pathways related to energy metabolism and inflammation affect brain regional metabolic status and cognitive function by metabolites crossing the BBB (Sheng et al., 2023; Zhao H. et al., 2025). Glycodeoxycholic acid is associated with cognitive impairment, with MRI white matter hyperintensities burden modifying this association (Kijpaisalratana et al., 2025). Abnormalities in bile acid metabolites parallel PET-measured Aβ and tau dynamics (Nabizadeh et al., 2024). Time-restricted feeding improves cognitive deficits and reduced neuroinflammation by enriching propionate, a metabolite derived from *Bifidobacterium pseudolongum,* and PET imaging confirmed that this propionate crosses the BBB (Zhao Y. et al., 2025).

Microbiota–brain structural associations have also been reported. Abundance of genera like *Bacteroides* and *Ruminococcus* correlates with hippocampal volume and cognitive performance, while *Akkermansia muciniphila* shows inverse correlations with brain amyloid, suggesting a potential protective role (Fan et al., 2025; Fan et al., 2023). Decreased *Ruminococcus* was also associated with impaired white matter integrity in the basal ganglia (Hung et al., 2023), while higher *Odoribacter* was associated with larger white-matter and hippocampal volume, and lower cerebrospinal fluid (CSF) volume (Xinxiu Liang et al., 2022). Furthermore, free water (FW) imaging revealed that increased FW content inversely correlated with butyrate-producing bacteria (Yamashiro et al., 2024). And gut-derived LPS-containing can cross the BBB, activate the Piezo1 channel on microglia, induce excessive synaptic pruning, and promote early AD pathology progression (Zhao X. et al., 2025).

### 5.2 Multiple sclerosis

Multiple sclerosis (MS) is a chronic degenerative disorder characterized by immune-mediated axonal damage and demyelination. Recent studies show significantly reduced gut microbiota diversity and altered taxa in MS patients, suggesting that gut dysbiosis is closely implicated in MS pathogenesis (Cox et al., 2021; Moles et al., 2021). MRI has become essential for the diagnosis and monitoring MS, especially in identifying white matter lesions and demyelinated areas, which correlates strongly with gut microbiota alterations. Significant microbial changes accompany disease progression in both experimental autoimmune encephalomyelitis (EAE) and cuprizone (CPZ) mouse models. Specifically, reduced SCFA-producing bacteria in MS patients correspond to increased MRI-visible lesion load and cerebral atrophy (Cox et al., 2021; Moles et al., 2021; Schwerdtfeger et al., 2025).

Gut microbiota modulate MS pathology by regulating the host immune system. SCFAs can modulate T cell differentiation, promoting the generation of regulatory T cells (Tregs) while suppressing pro-inflammatory Th17 cells (Miyake and Noto, 2021; Yousefi et al., 2022). Studies indicate that gut dysbiosis leads to impaired intestinal barrier function (“leaky gut”), allowing bacterial products like LPS to enter systemic circulation, activating immune responses in the CNS, and promoting inflammation and demyelination (Fan et al., 2024; Parodi and Kerlero de Rosbo, 2021). Furthermore, immune cells like IgA+ B cells specifically recognizing MS-associated gut microbiota can migrate to the CNS and correlate with active inflammatory activity (Pröbstel et al., 2020). Microbiota-modulating therapies, such as probiotics, dietary adjustments, and fecal microbiota transplantation (FMT), show potential to alleviate MS symptoms, partly by regulating immune function and metabolite production (Li et al., 2024; Yousefi et al., 2022).

### 5.3 Traumatic brain injury

Traumatic brain injury (TBI) is a common CNS injury involving multi-system interactions, while gut dysbiosis is increasingly implicated as an important mediator of neurological dysfunction (Rice et al., 2019). The MGBA’s neural, endocrine, and immune pathways play a critical role in TBI recovery and prognosis.

First, post-TBI gut dysbiosis is closely related to brain structural and functional impairments. TBI-induced white matter injury (WMI) involves oligodendrocyte lineage (OLCs). Gut microbiota influence OLC differentiation and proliferation by regulating peripheral immune cell infiltration. Notably, T-cell-deficient restored OLC proliferation and remyelination despite microbiota depletion, suggesting a key role for T cell–dependent microbiota–OLC regulatory axis (Shumilov et al., 2024). MRI findings of brain volume loss and decreased white matter connectivity further align with microbiome-driven structural remodeling (Davis et al., 2022; Davis Iv et al., 2025). Alterations in gut microbiota, specifically a decrease in Firmicutes and an increase in certain bacterial families within the Bacteroidetes and Proteobacteria phyla, Dysbiosis emerged rapidly, as early as 2 h, after CNS trauma with decreased Firmicutes and increased Bacteroidetes and Proteobacteria families, and predicted lesion volume and the severity of motor impairment (Kigerl et al., 2018; Nicholson et al., 2019).

Second, the gut-brain axis affects TBI recovery and prognosis through neural, endocrine, and immune pathways. Dysbiosis impairs gut barrier function and increases intestinal permeability, promoting the systemic spread of inflammatory factors that activate microglia and astrocytes in the CNS, thereby exacerbating neuroinflammation and neural damage (Oyovwi and Udi, 2025; You et al., 2025). Gut microbial metabolites like SCFAs regulate immune cell function and influence neural repair processes (Davis Iv et al., 2025). The neuroendocrine system, such as the growth hormone (GH) and insulin-like growth factor 1 (IGF-1) axis, is disrupted in TBI, linking gut dysfunction to impaired neuroregeneration (Venkatasamy et al., 2025; Yuen et al., 2020). Additionally, microbiome-regulated neuroimmune pathways (e.g., TLR4/MyD88/NF-κB, NLRP3 inflammasome) contribute to inflammatory and repair responses (Cai et al., 2025; Dong et al., 2024a).

Finally, neuroimaging, including high-resolution MRI and DTI, is essential for monitoring brain-gut-microbiome dynamics. Combined imaging with gut microbiota analysis and metabolomics reveals the relationship between the gut microbiome and brain functional impairment (Davis et al., 2022; Davis Iv et al., 2025). Single-cell RNA sequencing showed that SCFAs can modulate the microglial transcriptional profile, reduce neuroinflammation, and promote neuroprotection (Davis Iv et al., 2025). Furthermore, Fecal Microbiota Transplantation (FMT) has been shown to improve neurological deficits and structural damage after TBI, and reduce inflammation, suggesting therapeutic potential of microbiota modulation (Davis et al., 2022; Dong et al., 2024b).

### 5.4 Schizophrenia

Schizophrenia (SCZ) is a complicated and severe psychiatric disorder characterized by diverse clinical symptoms and cognitive impairment. Accumulating evidence has linked gut microbiota dysbiosis to structural and functional abnormalities in SCZ patients.

Neuroimaging studies reported that brain structure and FC are weakened in SCZ. Resting-state fMRI (rs-fMRI) studies found reduced intrinsic network connectivity correlated with changes in the abundance of specific gut bacterial genera (Peng et al., 2025). Similarly, structural MRI (sMRI) showed reduced gray matter volume in certain brain regions, and these regional abnormalities were linked to gut microbial composition (Li et al., 2021; Ma et al., 2020). A recent study has linked specific microbial signatures to region-specific structural changes. Specifically, increased *Eubacterium oxidoreducens* was associated with enlargement of the left lateral ventricle, while elevated abundances of Gordonibacter, Family XIII, and Parabacteroides were linked to volumetric alterations in the occipital and parietal, and increased SCZ risk (Ye et al., 2025). These findings suggested gut dysbiosis involved in SCZ pathology by modulating structural connectivity within vulnerable brain circuits.

Mechanistically, gut microbiota influence SCZ-related brain development and neuroplasticity via core MGBA pathways. A key SCZ feature, microglial dysfunction (Ben-Azu et al., 2023), is regulated by microbial metabolites and peripheral inflammation, with downstream effects on synaptic plasticity and neural network stability (Wu et al., 2025b; Wu et al., 2024).

Integrated imaging–microbiome analyses provide key insight into the regulatory role of microbes on brain network and clinical symptoms. Brain-gut-microbiota networks (BGMN) studies found that specific genera, like Faecalibacterium and Collinsella, were closely related to FC in the visual system, default mode network, and subcortical structures. These connectivity changes significantly correlated with cognitive performance and symptom severity in SCZ (Peng et al., 2025). Additionally, dysfunction of the brain’s glymphatic system (assessed via DTI-ALPS index) positively correlated with decreased gut microbial diversity and cognitive decline, suggesting gut microbiota may regulate neuroplasticity and brain reserve by affecting metabolic waste clearance (Wu et al., 2025b). Multi-omics integration analysis further supports a mechanistic role for microbial metabolites, as disturbances in GABA and tryptophan metabolism, were closely related to impaired brain FC and clinical symptoms (Wang Z. et al., 2024). The correlation between reduced microbial diversity, brain volume loss, and cognitive deficits in chronic medicated patients highlights the potential of microbiota-targeted therapeutic strategies to improve brain function and cognitive outcomes in SCZ (Ma et al., 2020; Wu et al., 2025a).

### 5.5 Autism spectrum disorder

Autism Spectrum Disorder (ASD) is a neurodevelopmental disorder characterized by social communication deficits, language impairments, and stereotyped repetitive behaviors. The pathological mechanisms of ASD are closely associated with the gut microbial community. Its core dysbiotic features include altered microbial composition, increased intestinal permeability, and enhanced inflammatory responses, all of which exhibit significant associations with neurodevelopmental abnormalities and behavioral symptoms in individuals with ASD (Hetta et al., 2025a).

Neuroimaging studies demonstrate that ASD patients exhibit widespread white matter abnormalities. Study based on DTI show uneven white matter development, initial over-myelination followed by reduced myelination with age, leading to impaired brain network function. Decreased axonal bundle integrity between key brain regions, such as the prefrontal cortex, cingulate gyrus, and amygdala, affecting interregional information transfer and consequently leading to social, cognitive, and behavioral deficits (Canada et al., 2025).

Genetic–microbiome–imaging evidence further supports a causal role for gut microbiota in ASD-related brain changes. A Mendelian randomization study reported that increased abundance of Lachnoclostridium predicts greater white matter volume in the left cerebellum, while increased Fusicatenibacter was associated with altered T2* signals in the right putamen, both changes linked to increased ASD risk. Further analysis revealed that the effect of gut microbiota on ASD appears to be largely mediated through structural changes in the brain (Ye et al., 2025). Additionally, microbial metabolites, such as tryptophan, have also been observed to be significantly associated with structural alterations and symptom severity (Aziz-Zadeh et al., 2025).

Experimental models further validate the regulatory role of gut microbiota in neural development. In mouse models, changes in gut microbiota composition were closely related to alterations in white matter fiber tracts on DTI. For instance, in a tuberous sclerosis mouse model, dietary modulation of gut microbiota significantly improved white matter structure and social behavior, suggesting gut microbiota influence brain connectivity by regulating neural myelination formation and repair (Hsieh et al., 2024). Zebrafish, as a convenient model for neuroimmune research, due to their transparent embryos and highly conserved enteric nervous system (ENS) structure, provide complementary mechanistic insights. Live imaging in zebrafish models shows real-time observation of interactions between gut immune cells and neurons, highlighting the importance of gut immune signaling in shaping neurodevelopmental trajectories (Andersen-Civil et al., 2023).

Mechanistically, ASD pathology is driven by coordinated dysregulation of the gut microbiota, ENS, and immune system, with core MGBA pathways mediating effects: gut dysbiosis-induced barrier dysfunction, immune activation, and microbial metabolite (e.g., SCFAs) perturbations regulate oligodendrocyte maturation, myelin stability, and neuroinflammation, contributing to white matter and neural network abnormalities (Canada et al., 2025; Murayama et al., 2025).

### 5.6 Depression

Depression is a common mental condition with multifactorial pathogenesis. Accumulating evidence highlights a bidirectional relationship between gut microbiota and the CNS via the MGBA.

In late-life depression (LLD), distinct gut microbial alterations, like increased *Enterobacter* and *Burkholderia,* correlate positively with depressive symptoms and negatively with gray matter volume in key brain regions involved in memory, somatosensory, and emotion regulation, suggesting gut dysbiosis may contribute to depression by affecting brain structure integrity (Tsai et al., 2022). And the abundance of *amycolatopsis* sp. *Hca4* modulates FC between the middle frontal gyrus and parahippocampal gyrus, thereby influencing working memory in a microbiota-dependent manner (Huang et al., 2025). Furthermore, LLD patients also exhibited reduced FC in emotion-regulation related brain regions, including the prefrontal cortex and caudate nucleus, and these connectivity pattern correlates with depression severity (Tsai et al., 2024). These results indicate that gut dysbiosis may contribute to depression via network-level disruptions.

Multi-omics and neuroimaging studies confirm this association between gut microbiota and brain structure/function. Children and adolescents with depression show altered gut microbiome accompanied by amino acid metabolism deficiencies, particularly decreased lysine, which induce depression-like behaviors by affecting prefrontal cortex metabolism (Teng et al., 2025). Additionally, specific genera like *Alistipes* correlate with gut metabolites (involving amino acid, vitamin B, and bile acid metabolism); these metabolites associate with functional alteration in the visual cortex, shaping cognitive and emotional dysfunction (Liu S. et al., 2025). Clinical trials further support intervention efficacy: probiotic intervention modulated gut microbiota composition, increased *Lactobacillus*, improved FC in prefrontal network, and alleviated depressive symptoms (Schaub et al., 2022; Yamanbaeva et al., 2023). Moreover, sleep deprivation-induced depressive phenotypes were similarly accompanied by gut dysbiosis and neuroinflammation, while astragalus supplementation restored SCFA levels, improving brain connectivity and inflammatory status (Jiao et al., 2025). These studies support a mechanistic role of microbial metabolites and immune signaling in depression pathogenesis.

Neuroimaging studies further reveal associations between gut microbiota and brain structure/function. Abnormal hippocampal FC correlates with increased abundance of pro-inflammatory gut bacteria (e.g., *Enterobacteriaceae*) in depressed patients (Lee et al., 2022). In geriatric depression, gut microbial diversity positively correlates with gray matter volume in the hippocampus and nucleus accumbens (Lee et al., 2022). Mendelian randomization analysis further supports a causal relationship between gut microbiota changes and brain function/structure metrics (Yang L. et al., 2025). Indoxyl sulfate (IS), a tryptophan-derived microbial product, may contribute to anxiety symptoms that is often accompanied by depression by activating resting-state FC in aversive-processing networks, including the subgenual anterior cingulate cortex (SCC-FC), bilateral anterior insula, right anterior mid-cingulate cortex, and right motor area (Brydges et al., 2021). Moreover, the interaction between Genus *Parabacteroides* and inflammatory factors is associated with static and dynamic low-frequency fluctuations in cerebellar Crus II in bipolar depression (Guo et al., 2024).

Together, the above evidence shows that gut microbiota mediate depression by affecting brain structural integrity, like the hippocampus and prefrontal cortex, and functional network organization, metabolic signaling pathways, and neuroinflammatory responses, providing converging neuroimaging and multi-omics evidence for the central role of the gut–brain axis in depressive pathophysiology.

## 6 Evidence from probiotic research

Probiotics multidimensionally regulate gut microecology and host physiological functions; for instance, they indirectly influence mood and cognition (e.g., alleviating anxiety-related behaviors) through the gut-brain axis, thereby exerting a crucial impact on physical health (Hetta et al., 2024). Randomized controlled trials exploring probiotics from a neuroimaging perspective indicate that modulation of the gut microbiota can influence brain function and structure, with effects observed in both task-based and resting-state fMRI.

In healthy populations, probiotic interventions (e.g., Ecologic®825, Puraflor) reduce activation in emotion- and cognition-related regions, including the amygdala, anterior cingulate cortex, and precuneus, while enhancing orbital frontal cortex activity, and altering resting-state connectivity in the salience and default mode networks, suggesting potential improvements in emotion regulation and cognitive control (Bagga et al., 2018; Rode et al., 2022; Tillisch et al., 2013). Similarly, a placebo-controlled probiotic trial of *Bifidobacterium longum* (*n* = 20 per group) also demonstrated that probiotic intervention modulated resting-state neural oscillations measured by magnetoencephalography (MEG). The observed MEG changes correlated with greater vitality and reduced mental fatigue during a social stress induction task (Wang et al., 2019).

In clinical populations, including patients with MDD or irritable bowel syndrome, probiotics (e.g., Vivomixx®, *Bifidobacterium longum* NCC3001) reduce activation in brain regions related to emotion processing (e.g., the putamen and amygdala activation) and improve FC in emotion-regulation networks, exhibiting a “normalizing” effect on abnormal neural function along the gut–brain axis (Schaub et al., 2022; Yamanbaeva et al., 2023). Although existing findings support the potential of probiotics to modulate CNS via the gut-brain axis, limitations remain. These include small sample sizes, heterogeneous strains and intervention protocols, and lack of long-term follow-up. Future studies will require more rigorously designed, longitudinal research integrating neuroimaging with multi-omics approach to better clarify mechanistic pathways and establish clinical application (Crocetta et al., 2024).

## 7 Harnessing neuroimaging to link gut microbiota to CNS disease treatments and future research

### 7.1 Application of neuroimaging in disease diagnosis and treatment monitoring

Advances in neuroimaging have significantly amplified its central role in the diagnosis and therapeutic monitoring of central nervous system (CNS) diseases. Neuroimaging markers combined with gut microbiota profiles not only aid early detection, mechanistic interpretation, but also enable dynamic assessment of therapeutic responses.

In early disease stages, fMRI and PET can reveal subtle changes in regional brain function and metabolism that parallel gut microbial changes. For example, in Parkinson’s disease, FDG-PET can identify motor- and cognition-related brain network patterns which may reflect underlying microbiota-associated pathophysiology, providing valuable markers for early diagnosis (Nakano et al., 2025). Similarly, MR-DTI can capture microstructural abnormalities in childhood Tourette syndrome, potentially linked to microbiota-related immune modulation (Xia et al., 2020).

Neuroimaging provides objective markers for dynamically evaluating therapeutic response by monitoring changes in brain structure, neurotransmission, and network connectivity. For instance, imaging of dopaminergic pathways and changes in brain network connectivity in PD can effectively guide medication adjustment and improve therapeutic outcomes (de Natale et al., 2021). Furthermore, multimodal imaging techniques like MRI and PET, combined with microbial metabolite data, hold promise for developing comprehensive biomarkers for precise diagnosis and longitudinal efficacy monitoring (van Oostveen and de Lange, 2021).

Neuroimaging also plays a key role in designing personalized therapeutic strategies. By combining brain structural and functional information with individual gut microbiota profiles, clinicians can develop targeted psychobiotic interventions, dietary strategies, or pharmacological treatments. Functional neuroimaging can identify dysregulated networks and, combined with gut microbiota regulatory mechanisms, allows comprehensive neuro–gut axis–based treatment plans (Nakano et al., 2025). Moreover, modern imaging techniques like photoacoustic imaging and magnetic resonance spectroscopy can monitor brain neuroinflammatory status and metabolic changes in real-time, improving medication adjustment and efficacy sensitivity (Cheng L. L. et al., 2025). In inflammatory and neurodegenerative diseases, like multiple sclerosis, imaging not only helps assess lesion distribution and extent but also captures regional trajectories, providing a basis for individualized medicine (Cagol et al., 2024; Zhang Y. et al., 2021). Furthermore, the integration of neuroimaging with AI is accelerating precision neuromedicine. Deep learning and analysis of imaging data can identify subtle biomarkers, enhance patient stratification, and improve prognostic accuracy (Tsampras et al., 2025).

In summary, neuroimaging markers combined with gut microbial features provide new perspectives and technical means for the early disease detection, treatment monitoring, and personalized therapeutic planning, thereby advancing precision management of CNS disorders.

### 7.2 Multi-omics integration and emerging imaging technologies

Driven by recent technological advances, research on the mechanisms linking gut microbiota to CNS diseases increasingly relies on multi-omics integration and emerging imaging techniques. Multi-omics approaches integrate microbiomic, metabolomic, immunomic, and neuroimaging data into systems biology framework, providing a comprehensive understanding of disease mechanisms. This multi-dimensional data integration compensates for the limitations of single-omics datasets and reveals the complex biological networks and dynamics of the gut-brain axis. In oncology and metabolic diseases, AI-based multi-omics fusion models significantly outperform single-modality approaches in disease classification, prognosis assessment, and treatment response prediction (Hatamikia et al., 2023; Wei et al., 2023). Similarly, radiomics combined with genomics and transcriptomics data can achieve spatiotemporal dynamic tracking of tumor heterogeneity, providing important guidance for precision diagnosis and treatment (Gevaert et al., 2020; Su et al., 2023). Furthermore, the development of multi-omics graph database systems further enhances large-scale data storage and cross-modal analysis, promoting efficient integration and mining of heterogeneous datasets (Thapa and Ali, 2021).

Emerging imaging technologies, such as single-cell imaging, functional connectome analysis, and machine learning-based approaches, greatly advance the in-depth analysis of gut-brain axis mechanisms. Single-cell imaging technology can map the spatial distribution of metabolites, proteins, and transcripts at cellular resolution, revealing key patterns of cellular heterogeneity and disease-related microenvironments (Lim et al., 2023). Spatial omics techniques, like spatial transcriptomics and multimodal mass spectrometry imaging, accurately localize cell types and metabolites within the tissue microenvironment, providing more precise insight into microbe-neural interactions along the gut-brain axis (Clarke et al., 2025; Hahn et al., 2025). Functional connectome analysis using fMRI, combined with machine learning algorithms, helps analyze dynamic changes in brain networks and their association with gut microbiota profiles in psychiatric disorders and cognitive impairment (Yang Q. et al., 2025; Zhang et al., 2025).

Machine learning and AI play increasing roles in multi-omics fusion and imaging analysis. Deep learning models automate the extraction and correlation analysis of high-dimensional biological and imaging features, improving the accuracy of disease risk prediction and classification (Ma et al., 2025; Zhao T. et al., 2025). For example, in complex diseases such as lung cancer and breast cancer, AI-assisted multimodal fusion models integrating clinical, imaging, and molecular data, have improved prognostic assessment and treatment decision-making (Singh et al., 2025; You et al., 2024). Additionally, digital twin technology combined with multi-omics and imaging datasets provides new clue for precision medicine by establishing individualized, dynamic disease models (Vallée, 2025). Continued advances in imaging resolution, analytical pipelines, and data standardization will further integrate multi-omics approaches with emerging imaging technologies, deepening mechanistic insights into the gut–brain axis and promoting early diagnosis, precise treatment, and personalized prognosis assessment of CNS diseases.

### 7.3 Future research challenges and prospects

The relationship between gut microbiota and CNS diseases, particularly brain structural and functional alterations detected by neuroimaging, has emerged as a major frontier in neuroscience and microbiome studies. Although substantial studies confirm that gut microbiota contribute to neurodegenerative diseases, psychiatric disorders, and brain injury via the gut-brain axis, the precise causal pathways and mechanistic details remain incompletely elucidated. A primary challenge for future research to clarify the causal relationship between gut microbial alterations and brain imaging alterations. Advances in multimodal MRI, PET, and microbiome-related metabolomics, provide powerful tools for revealing this causality. For example, Mendelian randomization studies have found causal associations between specific gut bacterial genera and iron-related brain imaging phenotypes, providing genetic evidence for microbiome effects on brain function and structure (Huang et al., 2024). Additionally, animal models, using germ-free approaches, microbiota transplantation, and high-resolution neuroimaging, help reveal molecular mechanisms of microbial regulation of brain microstructure and function (Montoro et al., 2022). However, the gut-brain axis is shaped by multiple factors, such as immune responses, metabolites, and CNS neuroinflammatory responses, highlighting the need for interdisciplinary, systematic research.

Promoting large-scale, multi-center, longitudinal clinical studies will be essential for future clinical application of these findings. Currently, the majority of studies are cross-sectional, limiting the capacity to reveal the dynamic interactions between gut microbiota changes, brain imaging features, and disease progression. Large, multi-center prospective cohorts with repeated sampling of microbiome, metabolome, and neuroimaging data will enable more robust causal inference and identification of biomarkers. For example, studies in multiple sclerosis intergrating microbiota profiles, immune status, and MRI assessment, have initially revealed associations between gut dysbiosis and central inflammation/demyelination (Moles et al., 2021; van Pamelen et al., 2020). Furthermore, longitudinal studies on AD and schizophrenia further demonstrate the potential of the gut-brain axis markers for early disease warning, therapeutic monitoring, and personalized treatment (Sheng et al., 2023; Wu et al., 2024). Future efforts should also strengthen multi-omics integration utilizing AI and machine learning, facilitating predictive modeling and accelerating the translation of basic science into clinical applications.

Future research should expand exploration of the gut-brain axis across a wider range of CNS diseases and therapeutic targets. Beyond AD, multiple sclerosis, and schizophrenia, conditions such as traumatic brain injury, depression, ASD, and vascular cognitive impairment also exhibit gut microbiota dysfunction accompanied by neuroimaging abnormalities. For instance, post-TBI gut dysfunction correlates with neuroinflammatory responses and neurological recovery, suggesting the gut-brain axis modulation as a potential therapeutic target (You et al., 2025). ASD research using zebrafish models combined with genetics and live imaging have revealed complex interactions between microbial signals, neural development, and enteric neuroimmune pathways (Andersen-Civil et al., 2023). Deeper mechanistic studies on microglial regulation, immune cell migration, and the role of inflammatory mediators, is particularly important for identifying new therapeutic strategies (Ben-Azu et al., 2023; Eva et al., 2023). Pharmacological or non-pharmacological interventions, including chemical drugs, exercise, dietary modification, probiotics, and FMT, have shown potential to modulate the gut-brain axis, both human and animal studies have found that probiotic supplementation may prevent brain atrophy as detected by MRI, providing new ideas for clinical intervention (Asaoka et al., 2022; Elsherbiny et al., 2025; Sanchez-Martinez et al., 2024; Yuan et al., 2025).

In summary, future research on gut microbiota and brain imaging should further clarify causal mechanisms, expand multi-center longitudinal studies, and broaden the disease spectrum. With multidisciplinary integration, technological innovation, and large-scale data support, the field will be profoundly reveal the fundamental role of the gut-brain axis in CNS diseases and promote precise diagnosis, treatment, and personalized intervention, ultimately improving neurological health and patient outcomes.

## 8 Conclusion

As research deepens into the relationship between gut microbiota and CNS diseases, increasing evidence indicates that gut microbiota profoundly influence brain structure and function through complex metabolic, immune, and neural signaling pathways, contributing to disease onset and progression. Rapid advances in this field not only deepens our understanding of neurological disease mechanisms but also provide new clue for diagnostic and therapeutic possibilities. The gut-brain axis has now reached a critical stage of multidisciplinary integration. Future research requires a deeper integration of theory and technology to achieve precise diagnosis, treatment, and personalized care for CNS disorders.
