# Supplementary material for: How gut microbiota contribute to neuropsychiatric disorders: evidence from neuroimaging studies
Source: Front Microbiol. 2026 Mar 4;17:1760096. doi: 10.3389/fmicb.2026.1760096 (PMC12996146; doi:10.3389/fmicb.2026.1760096)
Supplement: Supplementary file 1 [file Table_1.docx]

| Disorder | Shared Imaging Signatures | Disease-Specific Signatures | Contradictory Findings | Key References |
| --- | --- | --- | --- | --- |
| Alzheimer’s Disease (AD) | - DTI: Reduced white matter integrity (corpus callosum, cingulate gyrus)  - PET (TSPO): Increased neuroinflammation  - rs-fMRI: Reduced default mode network (DMN) functional connectivity (FC) | - PET: β-amyloid (Aβ) deposition and tau hyperphosphorylation (linked to reduced *Bacteroidetes*, abnormal bile acid metabolism)  - FW imaging: Increased gray matter free water (correlated with decreased butyrate-producing bacteria)  - MRI: Hippocampal atrophy (associated with *Akkermansia* muciniphila depletion) | - *Bacteroidete*s abundance: Most studies link reduction to Aβ positivity, but one cohort found no association (confounders: APOE genotype, diet)  - SCFA effects: Supplementation improves hippocampal volume in AD, but inconsistent in late-stage disease | Kojima et al., 2025; Yamashiro et al., 2024; Fan et al., 2023; Nabizadeh et al., 2024; X. Zhao et al., 2025 |
| Multiple Sclerosis (MS) | - DTI: Reduced fractional anisotropy (FA) in key white matter tracts  - PET (TSPO): Microglial activation  - MRI: Cerebral atrophy (linked to SCFA-producing bacteria depletion) | - T2/FLAIR MRI: Focal white matter demyelinating lesions (closely correlated with Th17/Treg imbalance from gut dysbiosis)  - Reduced SCFA producers (e.g., *Ruminococcus*) specific to MS-related neuroinflammation  - No other disorder shows direct dysbiosis-demyelination correlation | - SCFA levels: Some studies link depletion to lesion load, others find no association (modifiers: disease stage, immunotherapy)  - Gut barrier disruption: Consistently linked to neuroinflammation, but severity varies by patient subgroup | Cox et al., 2021; Schwerdtfeger et al., 2025; Moles et al., 2021; Fan et al., 2024; Pröbstel et al., 2020 |
| Traumatic Brain Injury (TBI) | - DTI: Impaired white matter connectivity  - sMRI: Regional gray matter volume loss  - PET: Post-injury neuroinflammation | - Acute sMRI: Rapid brain volume reduction (linked to *Firmicutes* depletion/*Bacteroidetes* overgrowth within 2 hours post-injury)  - DTI: Oligodendrocyte lineage dysfunction (mediated by T cell-microbiota crosstalk)  - FMT-reversible structural damage (unique to TBI gut-brain axis) | - Microbiota composition: Some studies link Proteobacteria overgrowth to motor impairment, others report no association (modifiers: injury severity)  - SCFA effects: Beneficial for neural repair in animal models, but human trial data are inconsistent | Davis et al., 2022; Davis Iv et al., 2025; Nicholson et al., 2019; Shumilov et al., 2024a; You et al., 2025 |
| Schizophrenia (SCZ) | - sMRI: Regional gray matter volume loss- rs-fMRI: Reduced DMN and prefrontal network FC- PET: Neuroinflammation (TSPO upregulation) | - sMRI: Left lateral ventricle enlargement (associated with *Eubacterium oxidoreducens* overgrowth)  - DTI-ALPS: Glymphatic system dysfunction (correlated with reduced microbial diversity)  - Unique link between *Faecalibacterium* depletion and visual system FC abnormalities | - Microbial diversity: Some studies link reduction to ventricular enlargement/cognitive impairment, others do not (modifiers: long-term medication)  - GABA metabolism: Disturbances linked to FC deficits in some cohorts but not others | Ye et al., 2025; Peng et al., 2025; H. Wu, B. Liu, et al., 2025; Ma et al., 2020; Z. Wang et al., 2024 |
| Autism Spectrum Disorder (ASD) | - DTI: Impaired white matter integrity (prefrontal cortex-amygdala tracts)  - rs-fMRI: Abnormal DMN and social cognition network FC  - PET: Mild neuroinflammation | - DTI: Uneven white matter development (early over-myelination → reduced myelination with age)  - T2* imaging: Altered signals in right putamen (linked to *Lachnoclostridiu*m and *Fusicatenibacter* overgrowth)  - Unique correlation between tryptophan metabolites and structural abnormalities | - Intestinal permeability: Linked to ASD symptoms, but not consistently associated with blood-brain barrier (BBB) dysfunction  - Microbial diversity: Some studies link reduction to social deficits, others report no correlation (confounders: age, diet) | Ye et al., 2025; Canada et al., 2025; Aziz-Zadeh et al., 2025; Hsieh et al., 2024; Hetta et al., 2025 |
| Depression | - sMRI: Hippocampal and prefrontal gray matter atrophy  - rs-fMRI: Reduced fronto-limbic FC  - PET: Mild neuroinflammation | - rs-fMRI: Indoxyl sulfate (IS)-mediated activation of aversive-processing networks (subgenual anterior cingulate cortex)  - Unique correlation between *Enterobacter* overgrowth/Alistipes depletion and fronto-hippocampal connectivity deficits  - Children/adolescents: Prefrontal cortex metabolic abnormalities from lysine deficiency (gut microbiota-driven) | - SCFA supplementation: Improves brain connectivity in some trials but not others (modifiers: depression subtype, intervention duration)  - Microbial diversity: Correlates with hippocampal volume in geriatric depression but not in young adults | Tsai et al., 2022; Tsai et al., 2024; Brydges et al., 2021; S. Liu et al., 2025; Jiao et al., 2025 |

Notes

Abbreviations: DTI (diffusion tensor imaging), PET (positron emission tomography), rs-fMRI (resting-state functional MRI), sMRI (structural MRI), FC (functional connectivity), DMN (default mode network), TSPO (translocator protein), FW (free water), BBB (blood-brain barrier), FMT (fecal microbiota transplantation).
